# Effect of irrigation and nitrogen application on grain amino acid composition and protein quality in winter wheat

**DOI:** 10.1371/journal.pone.0178494

**Published:** 2017-06-08

**Authors:** Panpan Zhang, Geng Ma, Chenyang Wang, Hongfang Lu, Shasha Li, Yingxin Xie, Dongyun Ma, Yunji Zhu, Tiancai Guo

**Affiliations:** 1 Agronomy College of Henan Agricultural University, Zhengzhou, Henan, China; 2 State Key Laboratory of Wheat and Maize Crop Science, Henan Agricultural University, Zhengzhou, Henan, China; Huazhong University of Science and Technology, CHINA

## Abstract

Water management and nitrogen application are critical factors in wheat grain yield and protein quality. This study aimed to evaluate the effect of irrigation and nitrogen application on the grain yield, protein content and amino acid composition of winter wheat. Field experiments were conducted in a split-plot design with three replications in high-yielding land on the North China Plain in 2012/2013, 2013/2014 and 2014/2015. Three irrigation treatments were examined in main plots: no irrigation, irrigation at jointing, and irrigation at jointing plus anthesis, while subplots were assigned to nitrogen treatment at four different rates: 0, 180, 240, 300 kg N ha^-1^, respectively. The results indicated that irrigation at jointing and at jointing plus anthesis improved grain yield by an average of 12.79 and 18.65% across three cropping seasons, respectively, compared with no irrigation. However, different irrigation treatments had no significant effect on grain protein content in any cropping season. Compared with no N treatment, 180, 240, and 300 kg N ha^-1^ N application significantly increased grain yield, by 58.66, 61.26 and 63.42% respectively, averaged over three cropping seasons. Grain protein and the total, essential and non-essential amino acid content significantly increased with increasing nitrogen application. Irrigation significantly improved the essential amino acid index (EAAI) and protein-digestibility-corrected amino acid score (PDCAAS) compared with no irrigation; however, N application decreased them by an average of 7.68 and 11.18% across three cropping seasons, respectively. EAAI and PDCAAS were positively correlated, however, they were highly negatively correlated with yield and grain protein content.

## Introduction

Owing to its ability to adapt to various environmental conditions and its utilization in a wide variety of food products, wheat is the most widely cultivated food crop in the world [1,2]. Achieving both high yield and grain quality is therefore a major goal in wheat production [3,4]. Wheat grain quality is determined by genetic and environmental factors: cultivar selection, climate conditions and management practices [5–7], mainly through their effects on protein content and composition [8,9].

Protein content and the balance of amino acids largely determine the nutrient quality of wheat grains. The essential amino acid (EAA) content in protein is lower than the non-essential amino acid (NAA) content, with glutamic acid (Glu) accounting for the majority of wheat grain protein [10,11]. Among EAA, lysine (Lys), tryptophan (Trp) and methionine (Met) are the most limiting in wheat grains, and as a result, have received much attention [12]. The content of Lys in wheat grains was found to average only 3.85, 3.37 and 3.15% in six emmer wheat, four old bread wheat and two modern bread wheat varieties, respectively [13]. Enhancing protein quality, especially the balance of amino acids in wheat grains, is therefore a critical issue in wheat production. Evaluation standards of the protein nutritive quality of amino acids have been set using the chemical score (CS) and protein-digestibility-corrected amino acid score (PDCAAS) [14,15], while the biological value is expressed by the essential amino acid index (EAAI). However, to the best of our knowledge, little has been done to determine the effect of crop management practices on EAAI and PDCAAS.

Irrigation is a key measure in improving grain yield in wheat production, especially in arid and semi-arid areas. With an increase in irrigation level, wheat grain yield is significantly improved [7]. Irrigation at critical stages of wheat growth such as early tillering, jointing, heading and flowering was found to result in higher grain yield through an increase in spike number, fertile florets and heavier single grain weight [16–18]. However, supplemental irrigation was also found to decrease the protein content of wheat grains [19]. Wang *et al*. [20] found that irrigation performed two or four times at the grain filling stage resulted in a significant decrease in the grain protein content. On the other hand, moderate water deficits during the grain filling stage were found to increase grain protein content, although a slight decrease in grain yield was also observed [21]. Despite these findings, few studies have evaluated the effect of irrigation on the amino acid composition of wheat grains.

Among the management practices employed in wheat production, nitrogen application is often found to be the most limiting factor in terms of yield and grain quality [22]. Increased nitrogen application, as well as optimization of the fertilizer type and timing of fertilization, is a common strategy aimed at increasing the spike or grain number per spike and improving the nitrogen content, and thereby yield and quality [23]. Moreover, the economic N application rate, which is based on the average yield potential and soil N test, matching nitrogen demand in time and space, is also important in terms of economical yield and quality [24]. Although nitrogen application has little effect on the ratio of EAA/total amino acid (TAA), it can significantly increase the content of TAA in wheat grains [25]. With increasing nitrogen, the percentage of Glu + glutamine (Gln) in the TAA was found to increase, while that of Lys and the ratio of cysteine (Cys) to Met decreased [26]. However, little is known about the effect of nitrogen rates on protein quality parameters in wheat grains.

Interactions between nitrogen application and irrigation have also been observed in wheat. Nitrogen uptake was greater under irrigation treatment compared with rain-sheltering conditions [27], and water use efficiency was significantly improved when nitrogen application was increased [28]. Bandyopadhyay *et al*. [29] also found that irrigation increased both water and nitrogen use efficiencies, resulting in an improvement in wheat grain yield. A factorial experiment in wheat showed that a higher grain yield but a lower protein content was observed with increasing irrigation across four nitrogen treatments, while under each individual irrigation treatment, grain protein content was increased with increasing nitrogen application [30]. The dilution effect of irrigation on grain protein content at higher nitrogen levels was greater than that at lower nitrogen rates [30,31]. These findings suggest that understanding the optimum regime of irrigation and nitrogen application is an important strategy in improving both grain yield and protein quality in winter wheat.

In China, wheat is a staple food, ranking second next to rice in terms of area and production [32]. Improving the protein quality of wheat grains through increased protein content and a better combination of amino acids is therefore urgently required. Effects of irrigation management, nitrogen fertilization application and the interaction between the two on wheat yield and protein content have already been researched in China [1,28]; however, little is known about the effects on grain amino acid composition and protein quality under field conditions. The objectives of this study, therefore, were to evaluate the effect of irrigation and nitrogen application management on wheat grain yield, amino acid composition and protein quality in winter wheat growing on the North China Plain. It was hypothesized that an appropriate increase in irrigation and nitrogen application rate would enhance the protein quality of the wheat grains.

## Materials and methods

### Field experiments

Long-term field experiments were commenced in 2010 at Wenxian (34°92′N, 112°99′E), Henan province, North China; a semi-arid area in the Huanghuai region for crop production of wheat-summer maize rotation. The land was owned by Pingan Seed Company Limited, and leased by Henan Agricultural University. We confirm that the field studies did not involve endangered or protected species.

Experiments were laid out in a split-plot design, with three irrigation treatments in the main plots (no irrigation, I0; irrigation at jointing, I1; irrigation at jointing plus anthesis, I2; irrigation of 750 m^3^ ha^–1^ each time) and four nitrogen rates in the sub-plots (0,180, 240 and 300 kg N ha^–1^; N0, N180, N240 and N300, respectively). Three replicates were performed for each treatment. All sub-plot treatments were randomized in each main plot treatment. Individual sub-plots were 6.1 m in length and 2.5 m wide, and consisted of 12 rows. Irrigation treatment involved uniform watering using movable pipelines, with the amount of water calculated using a water meter. Nitrogen fertilizer was applied as urea (46%), and phosphorus (P) and potassium (K) fertilizer as calcium superphosphate (15%) and potassium chloride fertilizer (60%) at rates of 150 (P_2_O_5_) and 120 (K_2_O) kg ha^-1^, respectively. At 1/2 urea, calcium superphosphate and potassium chloride fertilizer was sprayed onto the soil prior to soil preparation. Residual N fertilizer was then applied at the jointing stage in selected plots.

Yumai 49–198, a widely produced winter wheat cultivar in Huanghuai area, was used in this experiment. The sowing rate was 135 kg ha^-1^ with thinning to the recommended plant density in all subplots (approximately 55 plants per meter within a row) when most plants had 3–4 leaves. Protective management against pests and disease was carried out in all treatment plots to ensure healthy growth.

**Fig 1:**
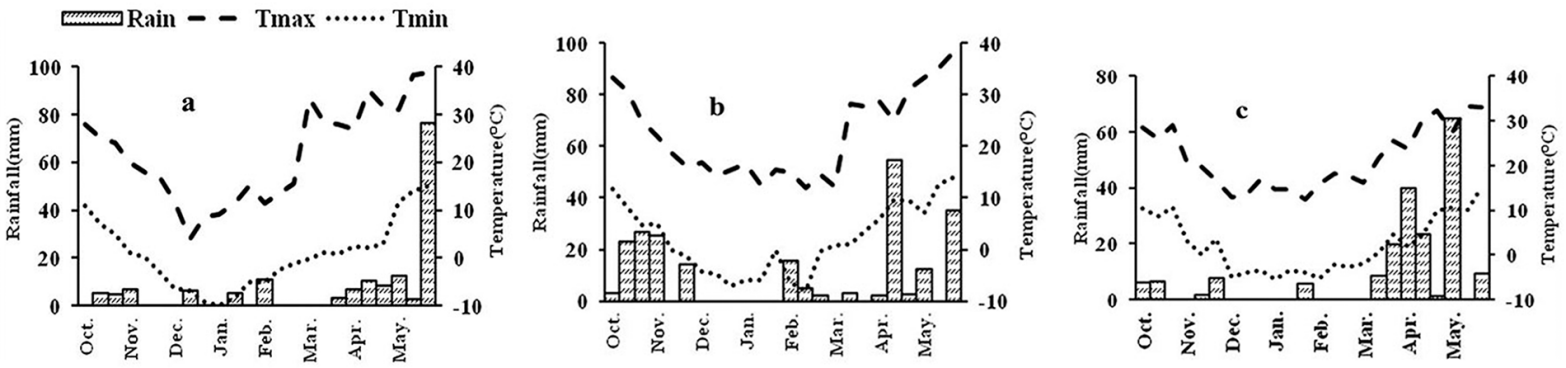
Rainfall distribution and maximum and minimum temperatures in the three cropping seasons: (a) 2012/2013, (b) 2013/2014 and (c) 2014/2015. Tmax: maximum temperature; Tmin: minimum temperature.

Wheat and soil samples were collected in the cropping seasons of 2012/2013, 2013/2014 and 2014/2015. Daily weather data for the three cropping seasons were obtained from a meteorological station located in the experimental field. The trends in temperature and rainfall relative to wheat growth are shown in Fig 1. Rainfall was limited and distributed mostly during the late stage of growth. The lowest daily maximum temperature (Tmax) was approximated to be 30°C and the average minimum temperature (Tmin) as 12.3°C during the wheat grain filling stage (May). Since soil nutrition could be affected by the different nitrogen rates, soil samples were collected before sowing at 0–30 cm from each plot under nitrogen treatment. Soil total N was measured by a semi-micro-Kjeldahl procedure. Available N was analyzed by the alkaline hydrolysis diffusion method, available P by the Olsen method, and available K using an atomic absorption spectrophotometer. Organic matter was determined by the K_2_Cr_2_O_7_-H_2_SO_4_ oxidation method, and pH was measured using an Orion Ionalyzer Model 901 pH meter in a 1:2.5 soil: water solution [33,34]. Soil chemical characteristics listed in Table 1 showed that, although significantly lower available N was observed in the N0 treatment in each cropping season, and lower available K content in 2014/2015, there were no significant differences in organic matter, total N, available P or pH among the different nitrogen treatments in the three cropping seasons.

**Table 1:** Initial chemical characteristics of the soil during the three cropping seasons.

Cropping Seasons	Treatments	Total N (g kg^-1^)	Available N (mg kg^-1^)	Available P (mg kg^-1^)	Available K (mg kg^-1^)	Organic matter (%)	pH
**2012/2013**	N0	0.91a	73.43c	22.07a	157.07a	16.98a	8.31a
	N180	0.95a	85.23b	23.60a	163.77a	17.12a	8.29a
	N240	1.04a	84.45b	24.18a	166.69a	17.55a	8.24a
	N300	0.97a	93.96a	24.23a	163.74a	17.85a	8.19a
	mean	0.97	84.27	23.52	162.82	17.37	8.26
**2013/2014**	N0	0.89a	70.28c	17.90a	102.69a	16.10a	8.28a
	N180	0.99a	89.64ab	17.64a	103.10a	16.21a	8.19a
	N240	1.03a	85.29b	17.95a	112.30a	16.35a	8.20a
	N300	0.95a	90.36a	17.41a	112.81a	16.60a	8.23a
	mean	0.97	83.89	17.73	107.73	16.32	8.23
**2014/2015**	N0	0.89a	72.59c	13.09a	116.77b	15.08a	8.32a
	N180	0.90a	79.28b	12.25a	120.25b	16.67a	8.20a
	N240	1.01a	84.54a	11.92a	146.97a	16.82a	8.26a
	N300	0.85a	89.56a	13.10a	158.36a	16.01a	8.21a
	mean	0.92	81.49	12.59	135.59	16.15	8.25

Note: Data represent the average value; values with different letters in the same column indicate a significant difference at the 5% level.

### Sample preparation and analysis of grain yield and protein quality

At maturity, wheat was hand-harvested in a 6 m^2^ area (2.4 m in length for 12 rows) in the middle of each plot. Grains were then threshed with a thresher and dried at 75°C until a constant weight was reached. Grain yield at corresponding moisture contents were then recorded and expressed against a standard moisture content of 13%.

Grain protein was calculated from the nitrogen content by multiplying by 5.7, and nitrogen content was measured using a nitrogen analyzer (Kjeltec 2300, FOSS, Sweden) according to the ICC Standard Method 105/2. The proportions of particular amino acids were determined using an L-8800 and L-8900 amino acid analysis meter (Hitachi High-Technologies Corporation) according to the ISO 13903–2005 method. EAA included threonine (Thr), valine (Val), isoleucine (Ile), leucine (Leu), phenylalanine (Phe), histidine (His), Met and Lys. Other amino acids comprised the NAA. EAAI was calculated by the EAA of protein in each sample and the EAA of reference egg protein [35], and PDCAAS was determined using the method described by Schaafsma *et al*. [36]:

$$\text{EAAI}={\left\{\left(\text{EAA}1\times \text{EAA}2\;\ldots \text{ EAAn}\right)\left[\text{samples}\right]/(\text{EAA}1\times \text{EAA}2\;\ldots \text{ EAAn})\left[\text{egg}\right]\right\}}^{1/\text{n}}$$

PDCAAS (%)=amino acid score (AAS)×true nitrogen digestibility (TD) (%)

$$\text{AAS}=\text{Content of the first limiting amino acid in the test protein }\left({\text{mg kg}}^{-1}\right)/\text{Content of the corresponding amino acid in the reference protein }\left({\text{mg kg}}^{-1}\right)$$

Here, the standard amino acid content in the protein of an adult (WHO/FAO/UNU, 2007) was used as the reference, and TD was 86% according to Tome [37].

### Statistical analysis

For all investigated parameters, analysis of variance (ANOVA) was performed using the SPSS statistical package based on a split-plot design. Irrigation and nitrogen application were taken as fixed factors, while cropping season was considered as a random factor due to unpredictable weather conditions. The F-test was used and when significant, differences were compared using the least significant difference (LSD) test at the 0.05 probability level. Correlation analysis was performed to determine the relationship among yield, protein content and quality across all treatments.

## Results

### Grain yield and amino acid composition

The results showed that the grain yield of winter wheat was significantly affected by irrigation and nitrogen application (Table 2). Grain yield was improved by irrigation and followed the trend I0 <I1 <I2. Compared with I0, I1 and I2 treatments increased the grain yield of winter wheat by 7.61 and 29.26% in 2012/2013, 16.72 and 25.65% in 2013/2014, and 12.86 and 15.61% in 2014/2015, which averaged 12.79 and 18.65% across all three cropping seasons. Nitrogen application significantly improved the grain yield of winter wheat. Compared with N0, N180, N240 and N300 increased the grain yield by an average of 58.66, 61.26 and 63.42%, respectively, across the three cropping seasons (Table 2). This result revealed a clear increase between N0 and N180 treatments, but additional nitrogen application did not increase the yield any further. However, the two-way interaction between irrigation and nitrogen application was not significant among the three cropping seasons. N240 under I2 in 2014/2015 and N0 under I0 in 2012/2013 exhibited the highest and lowest grain yields (9.11 and 3.71 t ha^-1^), respectively.

**Table 2:** Effects of irrigation and nitrogen application on grain yield and protein content in winter wheat in 2012/2013, 2013/2014 and 2014/2015, and interactions between irrigation and nitrogen application; summary of F significance from analysis of variance of the effects of main factors and interactions.

Treatment	Yield (t ha^-1^)			Protein (%)		
	2012/2013	2013/2014	2014/2015	2012/2013	2013/2014	2014/2015
**I0**	4.99b	6.16b	6.92c	14.76	15.33	14.33
**I1**	5.37b	7.19a	7.81b	13.34	14.91	14.33
**I2**	6.45a	7.74a	8.00a	14.22	14.89	14.35
**F-test**	**	**	**	ns	ns	ns
**N0**	4.58b	4.47b	4.80b	11.57c	14.20b	10.86c
**N180**	5.73a	7.84a	8.41a	14.32b	15.22a	15.17b
**N240**	6.03a	7.76a	8.55a	14.91ab	15.40a	15.54a
**N300**	6.08a	8.05a	8.54a	15.62a	15.34a	15.77a
**F-test**	**	**	**	**	**	**
**I0×N0**	3.71b	3.57b	4.32b	12.57b	14.37b	11.50b
**I0×N180**	4.94ab	6.67a	7.73a	15.33a	15.53a	15.10a
**I0×N240**	5.62a	7.34a	7.81a	15.37a	15.83a	15.10a
**I0×N300**	5.69a	7.07a	7.81a	15.77a	15.57a	15.60a
**F-test**	*	**	**	**	**	**
**I1×N0**	4.46b	4.60b	5.11b	11.50c	13.83b	10.67c
**I1×N180**	5.58a	8.39a	8.51a	12.40b	15.13a	15.03b
**I1×N240**	5.76a	7.63a	8.73a	13.93ab	15.30a	15.67a
**I1×N300**	5.70a	8.13a	8.87a	15.53a	15.37a	15.93a
**F-test**	**	**	**	**	**	**
**I2×N0**	5.56b	5.25b	4.98b	10.63b	14.40b	10.40c
**I2×N180**	6.65a	8.48a	8.99a	15.23a	15.00a	15.37b
**I2×N240**	6.72a	8.31a	9.11a	15.43a	15.07a	15.87a
**I2×N300**	6.86a	8.93a	8.93a	15.57a	15.10a	15.77a
**F-test**	*	**	**	**	**	**
**Grand mean**	5.60	7.03	7.57	14.11	15.04	14.33
**I×N (F-test)**	ns	ns	ns	**	ns	**
**CV (%)**	4.32	6.73	0.57	9.02	0.74	0.55

Note: ns, not significant at P < 0.05;

* Significant at P < 0.05;

** Significant at P < 0.01; Data in the same column with different letters indicate a significant difference at P < 0.05.

Compared with N0, nitrogen application significantly increased grain protein content in each cropping season. In contrast, no significant protein content response to irrigation was observed (Table 2). However, a two-way interaction between nitrogen application and irrigation on grain protein content was significant in 2012/2013 and 2014/2015. For example, in the cropping season of 2014/2015, no difference in grain protein content was observed among the three nitrogen treatments under I0, while under I1 and I2, the protein content of N180 was significantly lower than those of N240 and N300 respectively.

**Fig 2:**
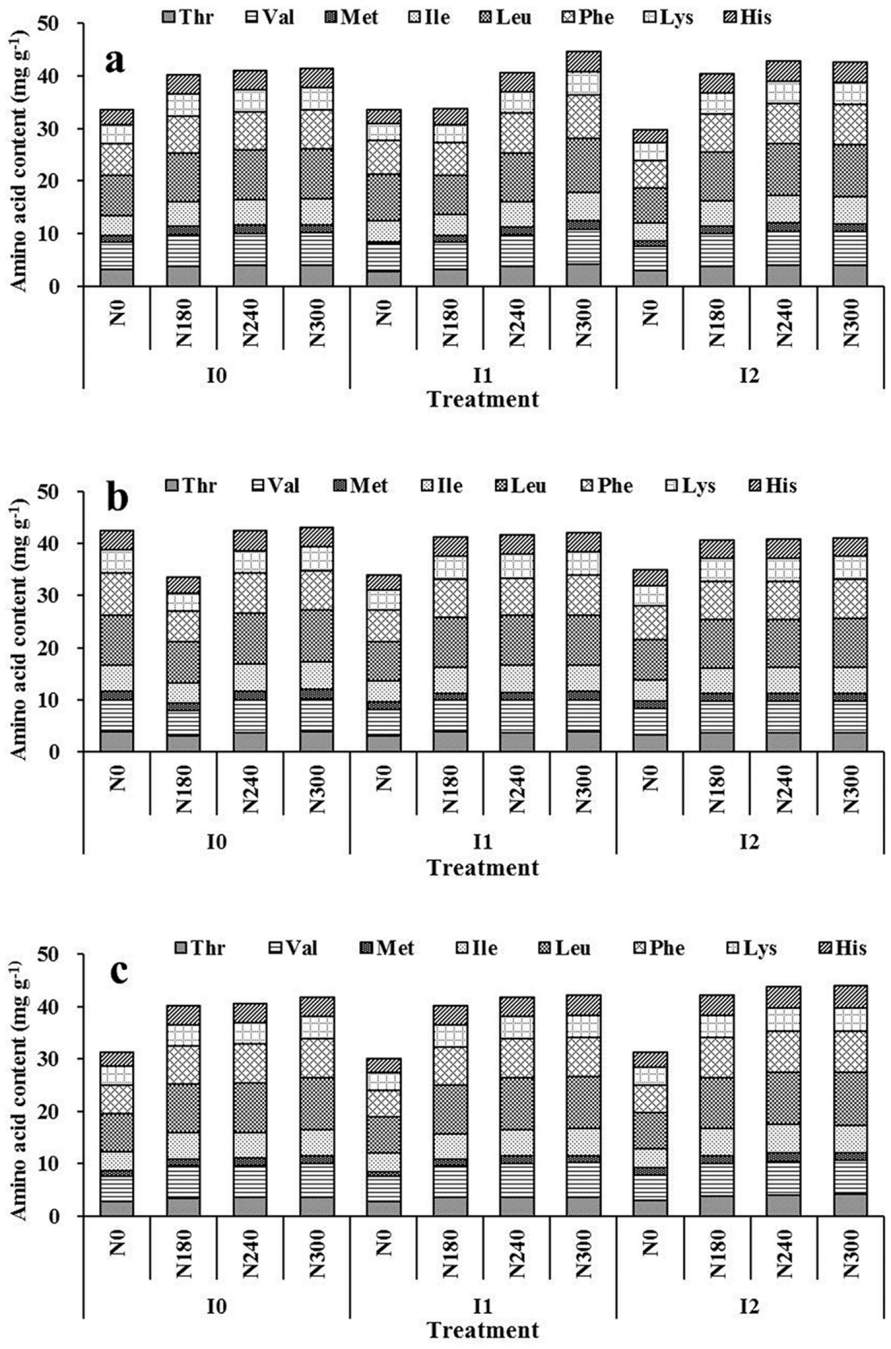
Analysis of variance of the effects of irrigation and nitrogen application on essential amino acid composition in 2012/2013(a), 2013/2014(b) and 2014/2015(c).

As shown in Table 3, TAA, EAA and NAA levels were not significantly affected by different irrigation regimes in 2012/2013 and 2014/2015, but all were significantly decreased by irrigation in 2013/2014. Furthermore, N application significantly increased all three parameters in the three cropping seasons. The highest values were observed in treatment N2, but no significant differences were observed between N2 and N3. The interaction of irrigation and nitrogen application had a significant effect on TAA, EAA and NAA content, and a similar trend was observed for grain protein content. The highest TAA, EAA and NAA levels were obtained at N300 under I2 in 2014/2015 (144.80, 43.90 and 100.90 mg g^-1^, respectively). Leu and Phe accounted for a large proportion of the EAA content. Lys levels ranged from 2.57 to 3.83 mg g^-1^, 2.90 to 3.77 mg g^-1^ and 2.77 to 4.13 mg g^-1^ in the three cropping seasons, respectively. A significant increase in EAA content was observed following higher nitrogen application in 2012/2013 and 2014/2015 (Fig 2). The ratio of EAA to TAA was affected by irrigation only in 2013/2014, but it was significantly decreased with increasing nitrogen application in the three cropping seasons. The interaction of irrigation and nitrogen application had a significant effect on the EAA/TAA ratio in all three cropping seasons, which varied from 30.11 to 32.58% (Table 4).

**Table 3:** Effects of irrigation and nitrogen application on TAA, EAA and NAA content in wheat grain in 2012/2013, 2013/2014 and 2014/2015, and interactions between irrigation and nitrogen application; summary of F significance from analysis of variance of the effects of main factors and interaction.

Treatment	TAA (mg·g^-1^)			EAA (mg·g^-1^)			NAA (mg·g^-1^)			EAA/TAA (%)		
	2012/2013	2013/2014	2014/2015	2012/2013	2013/2014	2014/2015	2012/2013	2013/2014	2014/2015	2012/2013	2013/2014	2014/2015
**I0**	127.29	128.98a	125.30	38.93	40.33a	38.46	88.36	88.65a	86.84	30.61	31.32b	30.75
**I1**	119.40	125.25b	126.18	38.08	39.73ab	38.55	81.40	85.53b	87.63	30.90	31.79a	30.66
**I2**	125.92	124.14b	131.30	38.85	39.36b	40.28	87.07	84.78b	91.02	31.02	31.74a	30.83
**F-test**	ns	**	ns	ns	*	ns	ns	**	ns	ns	**	ns
**N0**	114.38b	115.72c	96.69c	35.56c	37.08c	30.91c	78.82b	59.64c	65.78c	31.28a	32.04a	31.98a
**N180**	122.84b	121.34b	134.62b	38.06bc	38.44b	40.82b	84.79b	82.90b	93.80b	31.03ab	31.71a	30.33b
**N240**	136.02a	133.37a	138.33a	41.41ab	41.62a	42.03a	94.72a	91.74a	96.30a	30.54b	31.21b	30.39b
**N300**	140.23a	134.06a	140.73a	42.80a	42.07a	42.62a	97.43a	91.99a	98.11a	30.52b	31.39b	30.29b
**F-test**	**	**	**	**	**	**	**	**	**	*	*	**
**I0×N0**	107.63b	136.60a	99.43b	33.46b	42.47a	31.33b	74.17b	94.13a	68.10b	31.09a	31.09b	31.52a
**I0×N180**	131.43a	104.27b	131.57a	40.10a	33.43b	40.13a	91.33a	70.83b	91.43a	30.51b	32.07a	30.51b
**I0×N240**	134.83a	137.20a	132.83a	40.90a	42.40a	40.57a	93.93a	94.80a	92.27a	30.33b	30.91b	30.54b
**I0×N300**	135.27a	137.87a	137.37a	41.27a	43.03a	41.80a	94.00a	94.83a	95.57a	30.51b	31.21b	30.43b
**F-test**	**	**	**	**	**	**	**	**	**	**	**	**
**I1×N0**	144.10	103.40b	94.20c	43.43	33.90b	30.07c	100.67	69.50b	64.13c	30.15	32.78a	31.92a
**I1×N180**	106.43	130.50a	132.43b	33.77	41.27a	40.20b	72.67	89.23a	92.23b	31.74	31.63b	30.35b
**I1×N240**	131.00	133.27a	138.07a	40.53	41.63a	41.77a	90.80	91.63a	96.30a	31.15	31.24b	30.25b
**I1×N300**	146.07	133.83a	140.03a	44.60	42.10a	42.17a	101.47	91.73a	97.87a	30.55	31.46b	30.11b
**F-test**	ns	**	**	ns	**	**	ns	**	**	ns	**	**
**I2×N0**	91.40b	107.17b	96.43b	29.77b	34.87b	31.33c	61.63b	72.30b	65.10b	32.58a	32.54a	32.49a
**I2×N180**	130.67a	129.27a	139.87a	40.30a	40.63a	42.13b	90.37a	88.63a	97.73a	30.85b	31.43b	30.12b
**I2×N240**	142.23a	129.63a	144.10a	42.80a	40.83a	43.77a	99.43a	88.80a	100.33a	30.12b	31.50b	30.37b
**I2×N300**	139.37a	130.50a	144.80a	42.53a	41.10a	43.90a	96.83a	89.40a	100.90a	30.52b	31.49b	30.32b
**F-test**	**	**	**	**	**	**	**	**	**	**	**	**
**Grand mean**	124.23	126.13	127.60	38.62	39.81	39.10	85.61	86.32	88.50	30.84	31.62	30.75
**I×N (F-test)**	**	**	*	**	**	**	**	**	*	**	**	**
**CV (%)**	13.15	0.84	0.87	3.37	0.19	0.22	9.71	0.68	0.68	1.10	0.20	0.14

Note: TAA: total amino acid; EAA: essential amino acid; NAA: non-essential amino acid. ns, not significant at P < 0.05;

* Significant at P < 0.05;

** Significant at P < 0.01; Data in the same column with different letters indicate a significant difference at P < 0.05.

**Table 4:** Effects of irrigation and nitrogen application on EAAI and PDCAAS in wheat grain in 2012/2013, 2013/2014 and 2014/2015, and interactions between irrigation and nitrogen application; summary of F significance from analysis of variance of the effects of main factors and interactions.

Treatment	EAAI (%)			PDCAAS (%)		
	2012/2013	2013/2014	2014/2015	2012/2013	2013/2014	2014/2015
**I0**	58.70b	57.71	57.18b	51.99	53.00c	53.46b
**I1**	61.81a	57.98	57.72b	54.47	56.55a	54.18b
**I2**	60.21ab	57.12	61.58a	53.40	54.69b	56.54a
**F-test**	**	ns	**	ns	**	**
**N0**	61.01	64.29a	62.17a	55.74a	60.77a	61.15a
**N180**	58.54	54.76b	57.31b	51.98b	51.97b	52.64b
**N240**	61.34	56.18b	57.89b	53.51ab	53.24b	52.59b
**N300**	60.07	55.20b	57.94b	51.92b	53.01b	52.53b
**F-test**	ns	**	**	*	**	**
**I0×N0**	59.16	65.47a	58.47	53.30	62.57a	58.20a
**I0×N180**	58.34	46.82c	56.33	51.57	43.89c	51.06b
**I0×N240**	59.25	59.28b	57.01	51.78	51.13b	52.31b
**I0×N300**	58.04	59.28b	56.92	51.32	54.43b	52.27b
**F-test**	ns	**	ns	ns	**	**
**I1×N0**	61.60	65.81a	61.03a	54.76	61.75a	60.93a
**I1×N180**	59.45	59.11b	56.64b	54.63	56.82b	53.37b
**I1×N240**	63.65	59.11b	56.93b	55.20	59.54b	51.65bc
**I1×N300**	62.53	47.89c	56.27b	53.28	48.08c	50.77c
**F-test**	ns	**	**	ns	**	**
**I2×N0**	62.28	61.58a	67.00a	59.16a	57.98a	64.32a
**I2×N180**	57.81	58.33b	58.97b	49.74b	55.21a	53.48b
**I2×N240**	61.11	50.14c	59.74b	53.56b	49.05b	53.80b
**I2×N300**	59.63	58.43b	60.62b	51.15b	56.53a	54.54b
**F-test**	ns	**	**	*	**	**
**Grand mean**	60.24	57.60	58.83	53.29	54.75	54.73
**I×N (F-test)**	ns	**	**	ns	**	**
**CV (%)**	7.78	5.279349	2.06	15.06	5.80612382	2.43

Note: EAAI: essential amino acid index; PDCAAS: protein digestibility-corrected amino acid score; ns, not significant at P < 0.05;

* Significant at P < 0.05;

** Significant at P < 0.01; Data in the same column with different letters indicate a significant difference at P < 0.05.

### Protein quality evaluation

A significant increase in EAAI following irrigation was observed in 2012/2013 and 2014/2015, whereas increasing nitrogen application significantly decreased EAAI in 2013/2014 and 2014/2015 (Table 4). The interaction between irrigation and nitrogen application significantly affected EAAI in 2013/2014 and 2014/2015, due to the different effects of different nitrogen application treatments in each irrigation regime (Table 4). Irrigation and nitrogen application had a significant effect on PDCAAS, which increased with increasing irrigation, but decreased with increasing nitrogen application (Table 4). A two-way interaction between irrigation and nitrogen application was apparent for PDCAAS in 2013/2014 and 2014/2015, which ranged from 43.89 to 62.57% and 51.06 to 64.32%, respectively (Table 4).

### Correlations among grain yield, protein content and amino acid composition

**Fig 3:**
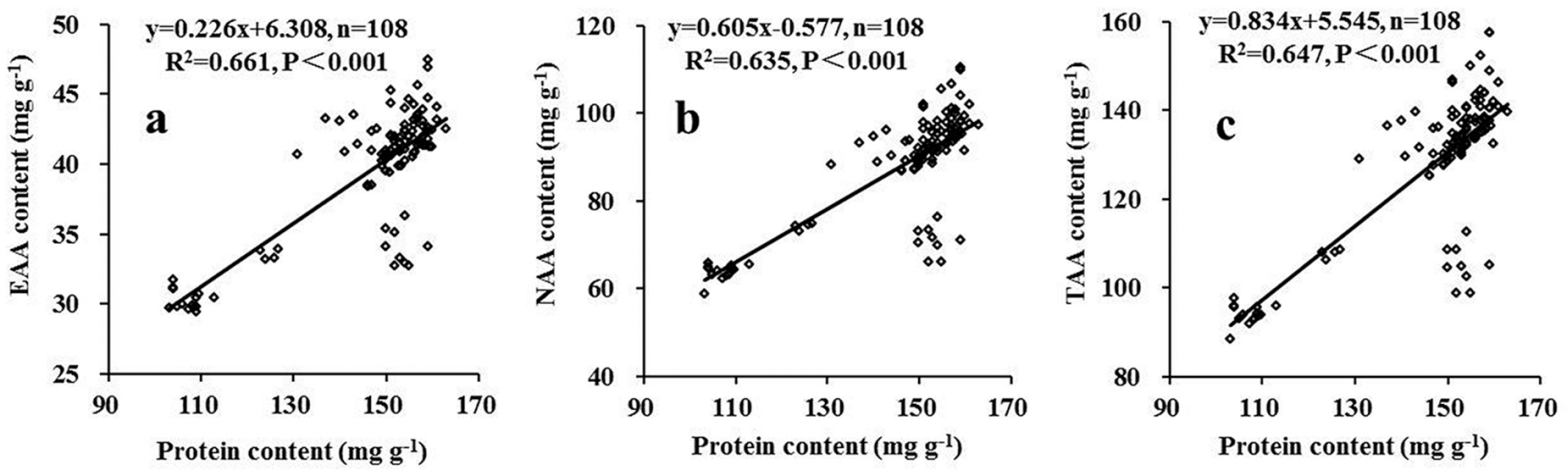
Relationships between grain protein content and (a) essential amino acids (EAA), (b) non-essential amino acids (NAA) and (c) total amino acids (TAA) content.

Significant correlations were observed among grain yield, protein content and amino acid composition (Table 5). Grain yield was positively correlated with protein, TAA and EAA content, but negatively with EAAI and PDCAAS (Pearson’s r = 0.511, 0.288, 0.296, -0.449 and -0.392, respectively). Close correlations were also found among protein content, TAA and EAA (0.805–0.991). Regression analysis further showed that the contents of EAA, NAA and TAA increased linearly with an increase in protein content (Fig 3). Similar slopes and correlation coefficients between grain protein and amino acid contents supported the view that higher EAA, NAA, and TAA amino acid contents are generally achieved under higher protein content. However, negative correlations were found between protein content and EAAI and PDCAAS (Pearson’s r = -0.362 and -0.579, respectively).

**Table 5:** Correlations among grain yield, protein content, amino acid content, EAAI and PDCAAS in the three cropping seasons.

	**Yield**	**Protein**	**TAA**	**EAA**	**EAAI**	**PDCAAS**
**Yield**	1					
**Protein**	0.511***	1				
**TAA**	0.288**	0.805***	1			
**EAA**	0.296**	0.813***	0.991***	1		
**EAAI**	-0.449***	-0.362***	0.229*	0.230*	1	
**PDCAAS**	-0.392***	-0.579***	-0.125	-0.108	0.782***	1

Note:

* Significant at P < 0.05;

** Significant at P < 0.01;

*** Significant at P < 0.001

## Discussion

### Grain protein and amino acid composition

The grain protein content, comprised of gliadins and glutenins (storage proteins), as well as albumins and globulins (metabolic proteins), is an important determinant of grain quality in wheat. It is intensively affected by nitrogen application and irrigation. Coventry *et al*. [38] reported that the highest protein content in wheat grains was obtained with the lowest of four irrigation treatments. With increasing irrigation, the crude protein content of wheat grains was decreased from 14 to 9% in two growing seasons [39]. Excess watering reduced glutenins, high molecular weight (HMW) glutenins and the ratio of HMW to low molecular weight (LMW) glutenin subunits [40,41]. The lower protein content in wheat grains as a result of irrigation is caused by yield dilution effects on grain protein [42,43]. However, in the present study, protein content was not significantly different in wheat grains in the three irrigation treatments, although irrigation significantly improved grain yield and the ratio of EAA/TAA in 2013/2014 (Tables 2 and 3). This result indicated that there was no effect of yield dilution in our experiment. This is because all experiments were carried out in the Huanghuai area of North China, where wheat growth is usually stressed by water shortage, especially at the jointing and anthesis stages of development. Here, irrigation performed both once and twice promoted nutritional absorption from the soil, significantly increasing grain yield; however, there was little effect on protein content (Table 3). This finding further suggests that irrigation at jointing plus anthesis would be optimal in this region.

N application can have a significant effect on protein and amino acid composition in wheat grains. Increasing nitrogen application can increase protein content in wheat grains significantly [44,45], mainly by stimulating the accumulation of gliadins and glutenins [46,47]. Zhang *et al*. [48] reported that protein, Leu, Phy and TAA content in wheat grains were all significantly increased following nitrogen application at two sites. In the present study, the protein and amino acid content were improved by nitrogen application (Tables 2 and 3). Furthermore, a N rate of 240 kg N ha^–1^ significantly increased protein, TAA, EAA and NAA content in wheat grain compared with 180 kg N ha^–1^, but no further increases were found for these traits on treatment with 300 kg N ha^–1^, indicating that a N rate of 240 kg ha^-1^ was already sufficient to satisfy N uptake requirements from soil, and maintain protein accumulation in wheat grain in the experimental conditions. In fact, NAA accumulated to a greater extent in wheat grains than did EAA when nitrogen was increased, due to high levels of glutamate (Glu) and alanine (Ala) in NAA. Additionally, although no nitrogen treatment resulted in a higher proportion of EAA relative to TAA, this ratio was not significantly different following nitrogen treatments of 180, 240 and 300 kg N ha^-1^, suggesting EAA and TAA varying to a similar extent in response to N application (Table 3).

### Protein quality parameters

The PDCAAS and CS of amino acids are used to evaluate the nutritive quality of protein. CS is affected by the wheat variety [13,49]; the CS of each EAA and the value of EAAI in wheat grains decrease significantly following insect infestation [50]. However, little is known about the effect of cultivation management on EAAI and PDCAAS under field conditions. In this study, EAAI and PDCAAS increased significantly with irrigation but decreased with N application (Fig 2). This suggests that irrigation improves the balance of amino acids, and therefore, the protein quality of wheat grains. Lys, the first limiting amino acid when calculating PDCAAS, significantly increased with increasing N application, consistent with the findings of Zhang *et al*. [48]. Michaelsen *et al*. [51] determined that the PDCAAS value of wheat for adults was 42–54%; however, in the current study, the range was 43.89–64.32%. This higher value was possibly caused by the fertile soil, suitable climate conditions or cultivar used.

### Correlation between yield and protein quality

Determining the fine-scale relationship between wheat grain yield and the concomitant grain protein content would provide valuable information on how to optimize cultivation management. Gursoy *et al*. [52] showed a negative correlation between wheat grain yield and protein content (r = -0.1177) under different tillage and residue management after cotton in three year field experiment. Furthermore, Li *et al*. [53] indicated that drought stress caused a reduction in yield but high grain protein content in 30 spring wheat varieties. However, in our study of different irrigation regimes and N application management, the correlation between yield and protein content was significantly positive (Table 5), mainly due to nitrogen management. Since nitrogen application is a crucial factor in wheat production, it results in a significant increase in yield and protein content compared to no N treatment [45,54], suggesting that both yield and protein content are increased to a comparable extent by nitrogen application. In addition, the nitrogen effect seems to be promoted by the irrigation regime [17,38], especially in semi-arid areas where the soil water is unable to meet the growth demands and irrigation therefore becomes important in terms of yield. This positive correlation was also observed by Nakano and Morita [55], who found that, compared to no N treatment, both yield and grain protein content were higher under application of 4 and 2 g m^2^ of nitrogen at tillering and jointing. Furthermore, Tosti and Guiducci [56] also reported a positive effect on grain yield and protein after incorporation of faba bean into the soil (to improve N availability for the cereal component). Thus, to a certain extent, N application is an efficient way of increasing the protein content without causing yield reductions.

In the present study, although the EAAI and PDCAAS of the wheat grains were positively correlated (r = 0.782), they were negatively correlated with yield and grain protein content (Table 5). This suggests that with increasing grain yield and protein content, accumulation of EAA, especially Lys, is less than that of total protein, decreasing the balance of the amino acid composition and the overall utilization of wheat grain protein. That is, a contradiction exists between grain yield and protein quality when attempting to improve grain yield via irrigation or fertilizer management. An efficient approach such as breeding of high-Lys wheat cultivars or determining optimal cultivation management is therefore needed in order to improve these essential amino acids and the overall quality of wheat grains.

## Conclusions

Both irrigation and nitrogen application significantly increased wheat grain yield. N application, but not irrigation, also had a significant and positive effect on grain protein content. The TAA, EAA and NAA content in grains also increased with increasing nitrogen, but no differences were observed under irrigation treatment in 2012/2013 and 2014/2015. In addition, EAAI and PDCAAS improved with irrigation but decreased with N application. Grain protein content was positively correlated with grain yield and contents of TAA and EAA, but negatively with EAAI and PDCAAS. Further analysis of the contradiction between yield and nutritional quality in wheat grains is now needed.

## Supporting information

S1 File Effects of irrigation and nitrogen application on 17 amino acids content in wheat grain in 2012/2013, 2013/2014 and 2014/2015, and interactions between irrigation and nitrogen application; summary of F significance from analysis of variance of the effects of main factors and interactions. Table A in S1 File: Asp, aspartic acid; Table B in S1 File: Thr, threonine; Table C in S1 File: Ser, serine; Table D in S1 File:Glu, glutamic acid; Table E in S1 File: Gly, glycine; Table F in S1 File: Ala, alanine; Table G in S1 File: Cys, cysteine; Table H in S1 File: Val, valine; Table I in S1 File: Met, methionine; Table J in S1 File: Ile, isoleucine; Table K in S1 File: Leu, leucine; Table L in S1 File: Tyr, tyrosine; Table M in S1 File: Phe, phenylalanine; Table N in S1 File: Lys, lysine; Table O in S1 File: His, histidine; Table P in S1 File: Arg, agrnine; Table Q in S1 File: Pro, proline.
(DOCX)

## Abbreviations

EAA: essential amino acid
EAAI: essential amino acid index
NAA: non-essential amino acid
PDCAAS: protein digestibility—corrected amino acid score
TAA: total amino acid
