# Supplementary material for: Effect of irrigation and nitrogen application on grain amino acid composition and protein quality in winter wheat
Source: PLoS One. 2017 Jun 8;12(6):e0178494. doi: 10.1371/journal.pone.0178494 (PMC5464558; doi:10.1371/journal.pone.0178494)
Supplement: S1 File — Table A in S1 File: Asp, aspartic acid; Table B in S1 File: Thr, threonine; Table C in S1 File: Ser, serine; Table D in S1 File:Glu, glutamic acid; Table E in S1 File: Gly, glycine; Table F in S1 File: Ala, alanine; Table G in S1 File: Cys, cysteine; Table H in S1 File: Val, valine; Table I in S1 File: Met, methionine; Table J in S1 File: Ile, isoleucine; Table K in S1 File: Leu, leucine; Table L in S1 File: Tyr, tyrosine; Table M in S1 File: Phe, phenylalanine; Table N in S1 File: Lys, lysine; Table O in S1 File: His, histidine; Table P in S1 File: Arg, agrnine; Table Q in S1 File: Pro, proline. (DOCX) [file pone.0178494.s001.docx]

Table A. Effects of irrigation and nitrogen application on Asp content in wheat grain in 2012/2013, 2013/2014 and 2014/2015, and interactions between irrigation and nitrogen application; summary of F significance from analysis of variance of the effects of main factors and interactions. Asp: aspartic acid.

|  |  |  |  |
| --- | --- | --- | --- |
| Treatment | Asp Content (mg·g^-1^) | | |
|  | 2012/2013 | 2013/2014 | 2014/2015 |
| I0 | 6.51a | 6.53a | 6.29c |
| I1 | 6.57a | 6.66a | 6.53b |
| I2 | 6.35a | 6.68a | 6.85a |
| F-test | ns | ns | ** |
|  |  |  |  |
| N0 | 5.87c | 6.12b | 5.12c |
| N180 | 6.26bc | 6.44a | 6.84b |
| N240 | 6.78ab | 6.96a | 7.07a |
| N300 | 7a | 6.96a | 7.19a |
| F-test | ** | ** | ** |
|  |  |  |  |
| I0×N0 | 5.57b | 7.07a | 5.17b |
| I0×N180 | 6.7a | 5.4b | 6.53a |
| I0×N240 | 6.87a | 6.77a | 6.63a |
| I0×N300 | 6.9a | 6.87a | 6.83a |
| F-test | * | ns | ** |
|  |  |  |  |
| I1×N0 | 7.07a | 5.6b | 5c |
| I1×N180 | 5.53a | 6.93a | 6.8b |
| I1×N240 | 6.5a | 7.07a | 7.13a |
| I1×N300 | 7.17a | 7.03a | 7.17a |
| F-test | ns | ** | ** |
|  |  |  |  |
| I2×N0 | 4.97b | 5.7b | 5.2b |
| I2×N180 | 6.53a | 7a | 7.2a |
| I2×N240 | 6.97a | 7.03a | 7.43a |
| I2×N300 | 6.93a | 6.97a | 7.57a |
| F-test | ** | ** | ** |
|  |  |  |  |
| Grand mean | 6.475 | 6.619 | 6.556 |
| I×N（F-test） | ** | ** | ** |
| CV（%） | 1.58 | 0.52 | 0.52 |

Table B. Effects of irrigation and nitrogen application on Thr content in wheat grain in 2012/2013, 2013/2014 and 2014/2015, and interactions between irrigation and nitrogen application; summary of F significance from analysis of variance of the effects of main factors and interactions. Thr: threonine.

|  |  |  |  |
| --- | --- | --- | --- |
| Treatment | Thr Content (mg·g^-1^) | | |
|  | 2012/2013 | 2013/2014 | 2014/2015 |
| I0 | 3.68a | 3.64a | 3.38b |
| I1 | 3.72a | 3.61ab | 3.39b |
| I2 | 3.68a | 3.52b | 3.76a |
| F-test | ns | * | ** |
|  |  |  |  |
| N0 | 3.36c | 3.4b | 2.88c |
| N180 | 3.57bc | 3.5a | 3.62b |
| N240 | 3.83ab | 3.7a | 3.73a |
| N300 | 4a | 3.75a | 3.8a |
| F-test | ** | ** | ** |
|  |  |  |  |
| I0×N0 | 3.2b | 3.93a | 2.87b |
| I0×N180 | 3.73a | 3.1b | 3.5a |
| I0×N240 | 3.87a | 3.73a | 3.53a |
| I0×N300 | 3.9a | 3.8a | 3.6a |
| F-test | ** | ns | ** |
|  |  |  |  |
| I1×N0 | 3.93a | 3.1b | 2.73b |
| I1×N180 | 3.13a | 3.8a | 3.53a |
| I1×N240 | 3.67a | 3.7a | 3.63a |
| I1×N300 | 4.13a | 3.83a | 3.67a |
| F-test | ns | ** | ** |
|  |  |  |  |
| I2×N0 | 2.93b | 3.17b | 3.03c |
| I2×N180 | 3.83a | 3.6a | 3.83b |
| I2×N240 | 3.97a | 3.67a | 4.03a |
| I2×N300 | 3.97a | 3.63a | 4.13a |
| F-test | ** | ** | ** |
|  |  |  |  |
| Grand mean | 3.689 | 3.589 | 3.508 |
| I×N（F-test） | ** | ** | NS |
| CV（%） | 1.42 | 0.52 | 0.50 |

Table C. Effects of irrigation and nitrogen application on Ser content in wheat grain in 2012/2013, 2013/2014 and 2014/2015, and interactions between irrigation and nitrogen application; summary of F significance from analysis of variance of the effects of main factors and interactions. Ser: serine.

|  |  |  |  |
| --- | --- | --- | --- |
| Treatment | Ser Content (mg·g^-1^) | | |
|  | 2012/2013 | 2013/2014 | 2014/2015 |
| I0 | 5.75a | 5.98a | 5.53b |
| I1 | 6a | 5.7b | 5.56b |
| I2 | 5.77a | 5.48c | 5.83a |
| F-test | ns | ** | ** |
|  |  |  |  |
| N0 | 5.29c | 5.25b | 4.41c |
| N180 | 5.58bc | 5.65a | 5.9b |
| N240 | 6.11ab | 5.99a | 6.09a |
| N300 | 6.38a | 5.99a | 6.16a |
| F-test | ** | ** | ** |
|  |  |  |  |
| I0×N0 | 5b | 6.4a | 4.53b |
| I0×N180 | 5.87a | 5.13c | 5.8a |
| I0×N240 | 6.03a | 6.4a | 5.87a |
| I0×N300 | 6.1a | 5.97b | 5.93a |
| F-test | * | ns | ** |
|  |  |  |  |
| I1×N0 | 6.6a | 4.57c | 4.23b |
| I1×N180 | 4.9a | 6.13ab | 5.83a |
| I1×N240 | 5.87a | 5.8b | 6.1a |
| I1×N300 | 6.63a | 6.3a | 6.07a |
| F-test | ns | ** | ** |
|  |  |  |  |
| I2×N0 | 4.27b | 4.77b | 4.47c |
| I2×N180 | 5.97a | 5.7a | 6.07b |
| I2×N240 | 6.43a | 5.77a | 6.3a |
| I2×N300 | 6.4a | 5.7a | 6.47a |
| F-test | ** | ** | ** |
|  |  |  |  |
| Grand mean | 5.839 | 5.719 | 5.639 |
| I×N（F-test） | ** | ** | * |
| CV（%） | 1.63 | 0.50 | 0.41 |

Table D. Effects of irrigation and nitrogen application on Glu content in wheat grain in 2012/2013, 2013/2014 and 2014/2015, and interactions between irrigation and nitrogen application; summary of F significance from analysis of variance of the effects of main factors and interactions. Glu: glutamic acid.

|  |  |  |  |
| --- | --- | --- | --- |
| Treatment | Glu Content (mg·g^-1^) | | |
|  | 2012/2013 | 2013/2014 | 2014/2015 |
| I0 | 42.19a | 43.37a | 43.75a |
| I1 | 43.99a | 41.5b | 43.93a |
| I2 | 41.62a | 41.14b | 43.85a |
| F-test | ns | ** | ns |
|  |  |  |  |
| N0 | 37.46c | 37.61c | 31.64c |
| N180 | 40.56bc | 40.43b | 46.56b |
| N240 | 45.19ab | 44.92a | 48.2a |
| N300 | 47.2a | 45.04a | 48.98a |
| F-test | ** | ** | ** |
|  |  |  |  |
| I0×N0 | 35.23b | 45.9a | 33.3b |
| I0×N180 | 43.6a | 34.43b | 46.43a |
| I0×N240 | 44.77a | 46.73a | 46.87a |
| I0×N300 | 45.17a | 46.4a | 48.4a |
| F-test | * | ns | ** |
|  |  |  |  |
| I1×N0 | 49.07a | 32.43b | 31.03c |
| I1×N180 | 34.63a | 43.7a | 46.13b |
| I1×N240 | 42.93a | 44.67a | 48.77a |
| I1×N300 | 49.33a | 45.2a | 49.8a |
| F-test | ns | ** | ** |
|  |  |  |  |
| I2×N0 | 28.07b | 34.5b | 30.6c |
| I2×N180 | 43.43a | 43.17a | 47.1b |
| I2×N240 | 47.87a | 43.37a | 48.97a |
| I2×N300 | 47.1a | 43.53a | 48.73ab |
| F-test | ** | ** | ** |
|  |  |  |  |
| Grand mean | 42.600 | 42.003 | 43.844 |
| I×N（F-test） | ** | ** | * |
| CV（%） | 1.89 | 0.50 | 0.45 |

Table E. Effects of irrigation and nitrogen application on Gly content in wheat grain in 2012/2013, 2013/2014 and 2014/2015, and interactions between irrigation and nitrogen application; summary of F significance from analysis of variance of the effects of main factors and interactions. Gly: glycine.

|  |  |  |  |
| --- | --- | --- | --- |
| Treatment | Gly Content (mg·g^-1^) | | |
|  | 2012/2013 | 2013/2014 | 2014/2015 |
| I0 | 5.27a | 5.28a | 5.31b |
| I1 | 5.6a | 5.26a | 5.38b |
| I2 | 5.31a | 5.3a | 5.58a |
| F-test | ns | ns | ** |
|  |  |  |  |
| N0 | 4.87c | 4.99c | 4.36c |
| N180 | 5.2bc | 5.08b | 5.66b |
| N240 | 5.64ab | 5.51a | 5.8a |
| N300 | 5.86a | 5.55a | 5.89a |
| F-test | ** | ** | ** |
|  |  |  |  |
| I0×N0 | 4.47b | 5.7a | 4.47b |
| I0×N180 | 5.47a | 4.33c | 5.5a |
| I0×N240 | 5.57a | 5.4b | 5.57a |
| I0×N300 | 5.57a | 5.7a | 5.7a |
| F-test | * | ns | ** |
|  |  |  |  |
| I1×N0 | 6.07a | 4.57b | 4.23b |
| I1×N180 | 4.67a | 5.4a | 5.63a |
| I1×N240 | 5.53a | 5.6a | 5.8a |
| I1×N300 | 6.13a | 5.47a | 5.87a |
| F-test | ns | ** | ** |
|  |  |  |  |
| I2×N0 | 4.07b | 4.7b | 4.37c |
| I2×N180 | 5.47a | 5.5a | 5.83b |
| I2×N240 | 5.83a | 5.53a | 6.03a |
| I2×N300 | 5.87a | 5.47a | 6.1a |
| F-test | ** | ** | ** |
|  |  |  |  |
| Grand mean | 5.392 | 5.281 | 5.425 |
| I×N（F-test） | ** | ** | * |
| CV（%） | 1.70 | 0.47 | 0.43 |

Table F. Effects of irrigation and nitrogen application on Ala content in wheat grain in 2012/2013, 2013/2014 and 2014/2015, and interactions between irrigation and nitrogen application; summary of F significance from analysis of variance of the effects of main factors and interactions. Ala: alanine.

|  |  |  |  |
| --- | --- | --- | --- |
| Treatment | Ala Content (mg·g^-1^) | | |
|  | 2012/2013 | 2013/2014 | 2014/2015 |
| I0 | 4.65a | 4.44ab | 4.58b |
| I1 | 4.88a | 4.36b | 4.65b |
| I2 | 4.63a | 4.61a | 4.79a |
| F-test | ns | * | ** |
|  |  |  |  |
| N0 | 4.3c | 4.32b | 3.79c |
| N180 | 4.59bc | 4.2a | 4.9b |
| N240 | 4.92ab | 4.68a | 4.97ab |
| N300 | 5.08a | 4.68a | 5.03a |
| F-test | ** | ** | ** |
|  |  |  |  |
| I0×N0 | 4b | 4.8a | 3.83b |
| I0×N180 | 4.8a | 3.53c | 4.8a |
| I0×N240 | 4.87a | 4.47b | 4.77a |
| I0×N300 | 4.93a | 4.97a | 4.9a |
| F-test | * | ns | ** |
|  |  |  |  |
| I1×N0 | 5.27a | 4.07b | 3.73c |
| I1×N180 | 4.13a | 4.3ab | 4.87b |
| I1×N240 | 4.87a | 4.83a | 5a |
| I1×N300 | 5.27a | 4.23ab | 5a |
| F-test | ns | ns | ** |
|  |  |  |  |
| I2×N0 | 3.63b | 4.1b | 3.8c |
| I2×N180 | 4.83a | 4.77a | 5.03b |
| I2×N240 | 5.03a | 4.73a | 5.13ab |
| I2×N300 | 5.03a | 4.83a | 5.2a |
| F-test | ** | ** | ** |
|  |  |  |  |
| Grand mean | 4.722 | 4.469 | 4.672 |
| I×N（F-test） | ** | ** | ns |
| CV（%） | 1.61 | 0.81 | 0.39 |

Table G. Effects of irrigation and nitrogen application on Cys content in wheat grain in 2012/2013, 2013/2014 and 2014/2015, and interactions between irrigation and nitrogen application; summary of F significance from analysis of variance of the effects of main factors and interactions. Cys: cysteine.

|  |  |  |  |
| --- | --- | --- | --- |
| Treatment | Cys Content (mg·g^-1^) | | |
|  | 2012/2013 | 2013/2014 | 2014/2015 |
| I0 | 1.79a | 1.67a | 1.27a |
| I1 | 1.58b | 1.4b | 1.34a |
| I2 | 1.64b | 1.33b | 1.28a |
| F-test | * | ** | ns |
|  |  |  |  |
| N0 | 1.57b | 1.34a | 1.16b |
| N180 | 1.61ab | 1.43a | 1.26b |
| N240 | 1.76a | 1.62a | 1.38a |
| N300 | 1.76a | 1.47a | 1.4a |
| F-test | * | ns | ** |
|  |  |  |  |
| I0×N0 | 1.57a | 1.73ab | 1.13b |
| I0×N180 | 1.87a | 1.4b | 1.27ab |
| I0×N240 | 1.87a | 2.03a | 1.33a |
| I0×N300 | 1.87a | 1.5b | 1.33a |
| F-test | ns | ns | ** |
|  |  |  |  |
| I1×N0 | 1.73a | 1.07b | 1.2b |
| I1×N180 | 1.33b | 1.57a | 1.27ab |
| I1×N240 | 1.57ab | 1.43a | 1.47a |
| I1×N300 | 1.7a | 1.53a | 1.43ab |
| F-test | ns | * | ns |
|  |  |  |  |
| I2×N0 | 1.4b | 1.23b | 1.13a |
| I2×N180 | 1.63ab | 1.33ab | 1.23a |
| I2×N240 | 1.83a | 1.4a | 1.33a |
| I2×N300 | 1.7ab | 1.37ab | 1.43a |
| F-test | ns | ns | ns |
|  |  |  |  |
| Grand mean | 1.672 | 1.467 | 1.297 |
| I×N（F-test） | * | ** | ns |
| CV（%） | 1.68 | 2.05 | 1.60 |

Table H. Effects of irrigation and nitrogen application on Val content in wheat grain in 2012/2013, 2013/2014 and 2014/2015, and interactions between irrigation and nitrogen application; summary of F significance from analysis of variance of the effects of main factors and interactions. Val: valine.

|  |  |  |  |
| --- | --- | --- | --- |
| Treatment | Val Content (mg·g^-1^) | | |
|  | 2012/2013 | 2013/2014 | 2014/2015 |
| I0 | 5.89a | 5.88a | 5.93a |
| I1 | 6.18a | 5.9a | 6.03a |
| I2 | 5.9a | 5.85a | 6.05a |
| F-test | ns | ns | ns |
|  |  |  |  |
| N0 | 5.49c | 5.47c | 4.87c |
| N180 | 5.83bc | 5.68b | 6.23b |
| N240 | 6.22ab | 6.13a | 6.41a |
| N300 | 6.42a | 6.23a | 6.5a |
| F-test | ** | ** | ** |
|  |  |  |  |
| I0×N0 | 5.17b | 6.07b | 4.87b |
| I0×N180 | 6.07a | 4.9c | 6.17a |
| I0×N240 | 6.13a | 6.13b | 6.23a |
| I0×N300 | 6.2a | 6.43a | 6.43a |
| F-test | * | ns | ** |
|  |  |  |  |
| I1×N0 | 6.67a | 5.1b | 4.83c |
| I1×N180 | 5.3a | 6.07a | 6.23b |
| I1×N240 | 6.1a | 6.23a | 6.47a |
| I1×N300 | 6.67a | 6.2a | 6.6a |
| F-test | ns | ** | ** |
|  |  |  |  |
| I2×N0 | 4.63b | 5.23b | 4.9c |
| I2×N180 | 6.13a | 6.07a | 6.3b |
| I2×N240 | 6.43a | 6.03a | 6.53a |
| I2×N300 | 6.4a | 6.07a | 6.47ab |
| F-test | ** | ** | ** |
|  |  |  |  |
| Grand mean | 5.992 | 5.878 | 6.003 |
| I×N（F-test） | ** | ** | ns |
| CV（%） | 1.37 | 0.41 | 0.39 |

Table I. Effects of irrigation and nitrogen application on Met content in wheat grain in 2012/2013, 2013/2014 and 2014/2015, and interactions between irrigation and nitrogen application; summary of F significance from analysis of variance of the effects of main factors and interactions. Met: methionine.

|  |  |  |  |
| --- | --- | --- | --- |
| Treatment | Met Content (mg·g^-1^) | | |
|  | 2012/2013 | 2013/2014 | 2014/2015 |
| I0 | 1.47a | 1.58a | 1.23b |
| I1 | 1.44a | 1.43b | 1.18b |
| I2 | 1.38a | 1.47b | 1.44a |
| F-test | ns | ** | ** |
|  |  |  |  |
| N0 | 1.28b | 1.4b | 1.04b |
| N180 | 1.34b | 1.4b | 1.29a |
| N240 | 1.54a | 1.58a | 1.4a |
| N300 | 1.54a | 1.58a | 1.39a |
| F-test | ** | ** | ** |
|  |  |  |  |
| I0×N0 | 1.2b | 1.57a | 0.93b |
| I0×N180 | 1.53a | 1.3b | 1.3a |
| I0×N240 | 1.57a | 1.7a | 1.27a |
| I0×N300 | 1.57a | 1.73a | 1.4a |
| F-test | * | ns | ** |
|  |  |  |  |
| I1×N0 | 1.57a | 1.33b | 0.93b |
| I1×N180 | 1.1b | 1.4ab | 1.13ab |
| I1×N240 | 1.5ab | 1.5a | 1.37a |
| I1×N300 | 1.6a | 1.47ab | 1.27a |
| F-test | ns | ns | * |
|  |  |  |  |
| I2×N0 | 1.07b | 1.3b | 1.27a |
| I2×N180 | 1.4a | 1.5a | 1.43a |
| I2×N240 | 1.57a | 1.53a | 1.57a |
| I2×N300 | 1.47a | 1.53a | 1.5a |
| F-test | * | ** | ns |
|  |  |  |  |
| Grand mean | 1.428 | 1.489 | 1.281 |
| I×N（F-test） | ** | ** | ns |
| CV（%） | 1.92 | 0.97 | 2.41 |

Table J. Effects of irrigation and nitrogen application on Ile content in wheat grain in 2012/2013, 2013/2014 and 2014/2015, and interactions between irrigation and nitrogen application; summary of F significance from analysis of variance of the effects of main factors and interactions. Ile: isoleucine.

|  |  |  |  |
| --- | --- | --- | --- |
| Treatment | Ile Content (mg·g^-1^) | | |
|  | 2012/2013 | 2013/2014 | 2014/2015 |
| I0 | 4.65a | 4.89a | 4.67b |
| I1 | 4.88a | 4.81ab | 4.69b |
| I2 | 4.66a | 4.74b | 4.88a |
| F-test | ns | * | ** |
|  |  |  |  |
| N0 | 4.2c | 4.37c | 3.62c |
| N180 | 4.53bc | 4.63b | 4.98b |
| N240 | 4.98ab | 5.12a | 5.14a |
| N300 | 5.2a | 5.13a | 5.23a |
| F-test | ** | ** | ** |
|  |  |  |  |
| I0×N0 | 3.93b | 5b | 3.7b |
| I0×N180 | 4.77a | 3.97c | 4.9a |
| I0×N240 | 4.93a | 5.27a | 4.93a |
| I0×N300 | 4.97a | 5.33a | 5.13a |
| F-test | * | ns | ** |
|  |  |  |  |
| I1×N0 | 5.27a | 4b | 3.53c |
| I1×N180 | 4a | 5a | 4.87b |
| I1×N240 | 4.8a | 5.17a | 5.13a |
| I1×N300 | 5.43a | 5.07a | 5.23a |
| F-test | ns | ** | ** |
|  |  |  |  |
| I2×N0 | 3.4b | 4.1b | 3.63b |
| I2×N180 | 4.83a | 4.93a | 5.17a |
| I2×N240 | 5.2a | 4.93a | 5.37a |
| I2×N300 | 5.2a | 5a | 5.33a |
| F-test | ** | ** | ** |
|  |  |  |  |
| Grand mean | 4.728 | 4.814 | 4.744 |
| I×N（F-test） | ** | ** | ns |
| CV（%） | 1.82 | 0.42 | 0.52 |

Table K. Effects of irrigation and nitrogen application on Leu content in wheat grain in 2012/2013, 2013/2014 and 2014/2015, and interactions between irrigation and nitrogen application; summary of F significance from analysis of variance of the effects of main factors and interactions. Leu: leucine.

|  |  |  |  |
| --- | --- | --- | --- |
| Treatment | Leu Content (mg·g^-1^) | | |
|  | 2012/2013 | 2013/2014 | 2014/2015 |
| I0 | 8.88a | 9.33a | 8.98b |
| I1 | 9.33a | 9.06b | 8.96b |
| I2 | 8.93a | 8.86c | 9.18a |
| F-test | ns | ** | ns |
|  |  |  |  |
| N0 | 8.1b | 8.37b | 7.01c |
| N180 | 8.67b | 8.82b | 9.48b |
| N240 | 9.56a | 9.5a | 9.77a |
| N300 | 9.87a | 9.63a | 9.91a |
| F-test | ** | ** | ** |
|  |  |  |  |
| I0×N0 | 7.53b | 9.73a | 7.2b |
| I0×N180 | 9.13a | 7.8b | 9.4a |
| I0×N240 | 9.43a | 9.83a | 9.53a |
| I0×N300 | 9.43a | 9.93a | 9.8a |
| F-test | * | ns | ** |
|  |  |  |  |
| I1×N0 | 10.17a | 7.6b | 6.87c |
| I1×N180 | 7.6a | 9.47a | 9.33b |
| I1×N240 | 9.27a | 9.53a | 9.77ab |
| I1×N300 | 10.3a | 9.63a | 9.87a |
| F-test | ns | ** | ** |
|  |  |  |  |
| I2×N0 | 6.6b | 7.77c | 6.97c |
| I2×N180 | 9.27a | 9.2ab | 9.7b |
| I2×N240 | 9.97a | 9.13b | 10a |
| I2×N300 | 9.87a | 9.33a | 10.07a |
| F-test | ** | ** | ** |
|  |  |  |  |
| Grand mean | 9.047 | 9.081 | 9.042 |
| I×N（F-test） | ** | ** | ns |
| CV（%） | 1.67 | 0.36 | 0.43 |

Table L. Effects of irrigation and nitrogen application on Tyr content in wheat grain in 2012/2013, 2013/2014 and 2014/2015, and interactions between irrigation and nitrogen application; summary of F significance from analysis of variance of the effects of main factors and interactions. Tyr: tyrosine.

|  |  |  |  |
| --- | --- | --- | --- |
| Treatment | Tyr Content (mg·g^-1^) | | |
|  | 2012/2013 | 2013/2014 | 2014/2015 |
| I0 | 2.38a | 1.94a | 1.45b |
| I1 | 2.43a | 1.92a | 1.47b |
| I2 | 2.2a | 1.79a | 2.52a |
| F-test | ns | ns | ** |
|  |  |  |  |
| N0 | 2.08b | 1.76b | 1.44b |
| N180 | 2.1b | 1.6b | 1.9a |
| N240 | 2.69a | 2.03a | 1.91a |
| N300 | 2.47a | 2.14a | 1.99a |
| F-test | ** | ** | ** |
|  |  |  |  |
| I0×N0 | 1.93b | 1.83b | 1.13b |
| I0×N180 | 2.5a | 1.13c | 1.5a |
| I0×N240 | 2.63a | 2.3ab | 1.53a |
| I0×N300 | 2.43a | 2.5a | 1.63a |
| F-test | ** | * | ns |
|  |  |  |  |
| I1×N0 | 2.6a | 1.87a | 1.07b |
| I1×N180 | 1.67b | 1.73a | 1.63a |
| I1×N240 | 2.8a | 2.13a | 1.57a |
| I1×N300 | 2.63a | 1.93a | 1.6a |
| F-test | * | ns | * |
|  |  |  |  |
| I2×N0 | 1.7b | 1.57b | 2.13a |
| I2×N180 | 2.13ab | 1.93a | 2.57a |
| I2×N240 | 2.63a | 1.67b | 2.63a |
| I2×N300 | 2.33ab | 2a | 2.73a |
| F-test | ns | * | ns |
|  |  |  |  |
| Grand mean | 2.333 | 1.883 | 1.811 |
| I×N（F-test） | * | ** | ns |
| CV（%） | 2.55 | 2.12 | 2.78 |

Table M. Effects of irrigation and nitrogen application on Phe content in wheat grain in 2012/2013, 2013/2014 and 2014/2015, and interactions between irrigation and nitrogen application; summary of F significance from analysis of variance of the effects of main factors and interactions. Phe: phenylalanine.

|  |  |  |  |
| --- | --- | --- | --- |
| Treatment | Phe Content (mg·g^-1^) | | |
|  | 2012/2013 | 2013/2014 | 2014/2015 |
| I0 | 6.94a | 7.27a | 6.9b |
| I1 | 7.35a | 7.09a | 6.84b |
| I2 | 6.94a | 7.22a | 7.08a |
| F-test | ns | ns | * |
|  |  |  |  |
| N0 | 6.3c | 6.8b | 5.28c |
| N180 | 6.83bc | 6.92b | 7.36b |
| N240 | 7.43ab | 7.44a | 7.52ab |
| N300 | 7.74a | 7.6a | 7.6a |
| F-test | ** | ** | ** |
|  |  |  |  |
| I0×N0 | 6.07b | 8a | 5.5b |
| I0×N180 | 7.13a | 5.9c | 7.27a |
| I0×N240 | 7.2a | 7.73ab | 7.33a |
| I0×N300 | 7.37a | 7.43b | 7.5a |
| F-test | * | ns | ** |
|  |  |  |  |
| I1×N0 | 7.57a | 6b | 5.13b |
| I1×N180 | 6.1a | 7.4a | 7.23a |
| I1×N240 | 7.53a | 7.17a | 7.47a |
| I1×N300 | 8.2a | 7.8a | 7.53a |
| F-test | ns | ** | ** |
|  |  |  |  |
| I2×N0 | 5.27b | 6.4b | 5.2b |
| I2×N180 | 7.27a | 7.47a | 7.57a |
| I2×N240 | 7.57a | 7.43a | 7.77a |
| I2×N300 | 7.67a | 7.57a | 7.77a |
| F-test | ** | ** | ** |
|  |  |  |  |
| Grand mean | 7.078 | 7.192 | 6.939 |
| I×N（F-test） | * | ** | ns |
| CV（%） | 1.89 | 0.56 | 0.46 |

Table N. Effects of irrigation and nitrogen application on Lys content in wheat grain in 2012/2013, 2013/2014 and 2014/2015, and interactions between irrigation and nitrogen application; summary of F significance from analysis of variance of the effects of main factors and interactions. Lys: lysine.

|  |  |  |  |
| --- | --- | --- | --- |
| Treatment | Lys Content (mg·g^-1^) | | |
|  | 2012/2013 | 2013/2014 | 2014/2015 |
| I0 | 4.01a | 4.23b | 3.98b |
| I1 | 4.08a | 4.4a | 4.02b |
| I2 | 3.94a | 4.26b | 4.19a |
| F-test | ns | * | ** |
|  |  |  |  |
| N0 | 3.74c | 4.06c | 3.47c |
| N180 | 3.87bc | 4.13bc | 4.18b |
| N240 | 4.18ab | 4.49b | 4.28ab |
| N300 | 4.24a | 4.51a | 4.33a |
| F-test | * | ** | ** |
|  |  |  |  |
| I0×N0 | 3.5b | 4.43b | 3.5b |
| I0×N180 | 4.13a | 3.57d | 4.03a |
| I0×N240 | 4.17a | 4.23c | 4.13a |
| I0×N300 | 4.23a | 4.7a | 4.27a |
| F-test | ns | ns | ** |
|  |  |  |  |
| I1×N0 | 4.43a | 3.87b | 3.4b |
| I1×N180 | 3.5a | 4.5a | 4.2a |
| I1×N240 | 4.03a | 4.77a | 4.23a |
| I1×N300 | 4.33a | 4.47a | 4.23a |
| F-test | ns | ** | ** |
|  |  |  |  |
| I2×N0 | 3.3b | 3.87b | 3.5c |
| I2×N180 | 3.97a | 4.33a | 4.3b |
| I2×N240 | 4.33a | 4.47a | 4.47ab |
| I2×N300 | 4.17a | 4.37a | 4.5a |
| F-test | * | ** | ** |
|  |  |  |  |
| Grand mean | 4.008 | 4.297 | 4.064 |
| I×N（F-test） | ** | ** | ns |
| CV（%） | 1.48 | 0.53 | 0.44 |

Table O. Effects of irrigation and nitrogen application on His content in wheat grain in 2012/2013, 2013/2014 and 2014/2015, and interactions between irrigation and nitrogen application; summary of F significance from analysis of variance of the effects of main factors and interactions. His: histidine.

|  |  |  |  |
| --- | --- | --- | --- |
| Treatment | His Content (mg·g^-1^) | | |
|  | 2012/2013 | 2013/2014 | 2014/2015 |
| I0 | 3.42a | 3.52a | 3.4b |
| I1 | 3.61a | 3.43a | 3.44b |
| I2 | 3.43a | 3.45a | 3.71a |
| F-test | ns | ns | ** |
|  |  |  |  |
| N0 | 3.09b | 3.22b | 2.74c |
| N180 | 3.41ab | 3.35b | 3.69b |
| N240 | 3.67a | 3.66a | 3.78ab |
| N300 | 3.78a | 3.63a | 3.86a |
| F-test | ** | ** | ** |
|  |  |  |  |
| I0×N0 | 2.87b | 3.73a | 2.77b |
| I0×N180 | 3.6a | 2.9b | 3.57a |
| I0×N240 | 3.6a | 3.77a | 3.6a |
| I0×N300 | 3.6a | 3.67a | 3.67a |
| F-test | * | ns | ** |
|  |  |  |  |
| I1×N0 | 3.83a | 2.9b | 2.63b |
| I1×N180 | 3.03a | 3.63a | 3.67a |
| I1×N240 | 3.63a | 3.57a | 3.7a |
| I1×N300 | 3.93a | 3.63a | 3.77a |
| F-test | ns | ** | ** |
|  |  |  |  |
| I2×N0 | 2.57b | 3.03b | 2.83c |
| I2×N180 | 3.6a | 3.53a | 3.83b |
| I2×N240 | 3.77a | 3.63a | 4.03a |
| I2×N300 | 3.8a | 3.6a | 4.13a |
| F-test | ** | ** | ** |
|  |  |  |  |
| Grand mean | 3.486 | 3.467 | 3.517 |
| I×N（F-test） | * | ** | * |
| CV（%） | 1.83 | 0.60 | 0.50 |

Table P. Effects of irrigation and nitrogen application on Arg content in wheat grain in 2012/2013, 2013/2014 and 2014/2015, and interactions between irrigation and nitrogen application; summary of F significance from analysis of variance of the effects of main factors and interactions. Arg: agrnine.

|  |  |  |  |
| --- | --- | --- | --- |
| Treatment | Arg Content (mg·g^-1^) | | |
|  | 2012/2013 | 2013/2014 | 2014/2015 |
| I0 | 6.48a | 6.4a | 5.95b |
| I1 | 6.65a | 6.25ab | 6.03b |
| I2 | 6.27a | 6.14b | 6.65a |
| F-test | ns | * | ** |
|  |  |  |  |
| N0 | 5.81b | 5.95b | 4.93c |
| N180 | 6.19b | 5.95b | 6.48b |
| N240 | 6.91a | 6.57a | 6.63ab |
| N300 | 6.94a | 6.58a | 6.8a |
| F-test | ** | ** | ** |
|  |  |  |  |
| I0×N0 | 5.37b | 7a | 4.87b |
| I0×N180 | 6.8a | 5.23c | 6.17a |
| I0×N240 | 6.9a | 6.77b | 6.27a |
| I0×N300 | 6.83a | 6.6b | 6.5a |
| F-test | * | ns | ** |
|  |  |  |  |
| I1×N0 | 7.27a | 5.43b | 4.73b |
| I1×N180 | 5.33a | 6.33a | 6.37a |
| I1×N240 | 6.77a | 6.5a | 6.47a |
| I1×N300 | 7.23a | 6.73a | 6.57a |
| F-test | ns | ** | ** |
|  |  |  |  |
| I2×N0 | 4.8b | 5.43b | 5.2b |
| I2×N180 | 6.43a | 6.3a | 6.9a |
| I2×N240 | 7.07a | 6.43a | 7.17a |
| I2×N300 | 6.77a | 6.4a | 7.33a |
| F-test | ** | ** | ** |
|  |  |  |  |
| Grand mean | 6.464 | 6.264 | 6.211 |
| I×N（F-test） | ** | ** | ns |
| CV（%） | 1.72 | 0.51 | 0.65 |

Table Q. Effects of irrigation and nitrogen application on Pro content in wheat grain in 2012/2013, 2013/2014 and 2014/2015, and interactions between irrigation and nitrogen application; summary of F significance from analysis of variance of the effects of main factors and interactions. Pro: proline.

|  |  |  |  |
| --- | --- | --- | --- |
| Treatment | Pro Content (mg·g^-1^) | | |
|  | 2012/2013 | 2013/2014 | 2014/2015 |
| I0 | 13.35a | 13.05a | 12.72b |
| I1 | 13.7a | 12.48b | 12.74b |
| I2 | 13.28a | 12.31b | 13.67a |
| F-test | ns | ** | * |
|  |  |  |  |
| N0 | 11.59b | 11.3c | 8.92b |
| N180 | 12.71b | 12.1b | 14.31a |
| N240 | 14.72a | 13.47a | 14.26a |
| N300 | 14.76a | 13.59a | 14.68a |
| F-test | ** | ** | ** |
|  |  |  |  |
| I0×N0 | 11.03b | 13.7a | 9.67b |
| I0×N180 | 13.73a | 10.23b | 13.43a |
| I0×N240 | 14.43a | 13.93a | 13.43a |
| I0×N300 | 14.2a | 14.33a | 14.33a |
| F-test | ** | ns | ** |
|  |  |  |  |
| I1×N0 | 15ab | 9.9b | 8.9b |
| I1×N180 | 10.47b | 13.13a | 13.7a |
| I1×N240 | 13.97ab | 13.6a | 14a |
| I1×N300 | 15.37a | 13.3a | 14.37a |
| F-test | ns | ** | ** |
|  |  |  |  |
| I2×N0 | 8.73b | 10.3b | 8.2b |
| I2×N180 | 13.93a | 12.93a | 15.8a |
| I2×N240 | 15.77a | 12.87a | 15.33a |
| I2×N300 | 14.7a | 13.13a | 15.33a |
| F-test | ** | ** | ** |
|  |  |  |  |
| Grand mean | 13.444 | 12.614 | 13.042 |
| I×N（F-test） | ** | ** | ** |
| CV（%） | 1.98 | 0.55 | 1.05 |
